# Modelling the Effects of Selection Temperature and Mutation on the Prisoner’s Dilemma Game on a Complete Oriented Star

**DOI:** 10.1371/journal.pone.0107417

**Published:** 2014-10-14

**Authors:** Jianguo Ren, Yonghong Xu

**Affiliations:** 1 Department of Computer Science, Jiangsu Normal University, Xuzhou, China; 2 Department of Live Science, Jiangsu Normal University, Xuzhou, China; Kyushu University, Japan

## Abstract

This paper models the prisoner’s dilemma game based on pairwise comparison in finite populations on a complete oriented star (COS). First, we derive a linear system on a COS for calculating the corresponding fixation probabilities that imply dependence of the selection temperature and mutation. Then we observe and analyze the effects of two parameters on fixation probability under different population sizes. In particular, it is found through the experimental results that (1) high mutation is more sensitive to the fixation probability than the low one when population size is increasing, while the opposite is the case when the number of cooperators is increasing, and (2) selection temperature demotes the fixation probability.

## Introduction

As a standard approach to describing the evolutionary dynamics of population, evolutionary game theory has drawn considerable attentions ([1], [2], [3], [4], [5], [6], [7], [8], [9]). In this context, an ideal assumption is that individuals interact randomly with each other in large, well-mixed population. In real world, however, the organization of population is usually highly structured rather than uniform, where the dynamics between individuals depend on both the strategy and the population configuration ([10], [11], [12], [13], [14], [15], [16], [17], [18]). The population configuration can be modeled by a weighted digraph. A *complete oriented star* (COS) is a complete bipartite digraph, where one partition consists of a single vertex known as the central vertex, and the other is a collection of vertices known as the peripheral vertices. COSs are a popular class of network topologies ([19], [20]) and have a wide-ranging applied background, such as computer network topology and a social organization, where the leader and employees can be seen as the central vertex and the peripheral vertices, respectively. The communications between them can be represented by the weight. To our knowledge, the evolutionary game with mutation based on the pairwise comparison process with COS structure has yet to be investigated.

Taking the prisoner’s dilemma ([21], [22], [23], [24]) as an example, this paper addresses the evolutionary game of population with COS structure, where the mutation is based on pairwise comparison ([25], [26], [27]). First, we derive a linear system for calculating the fixation probabilities. Then we observe and analyze such phenomena appeared in the game as the effects of selection temperature and mutation rates and population size on fixation probability.

The organization of this paper is as follows. In the next section, we present preliminary knowledge to support our study. In section 3, we derive analytic solutions of fixation probabilities by converting our task into a linear system on a COS. In Section 4, we give some simulations. Finally, we end the paper in Section 5.

## Preliminary Knowledge

**Figure 1:**
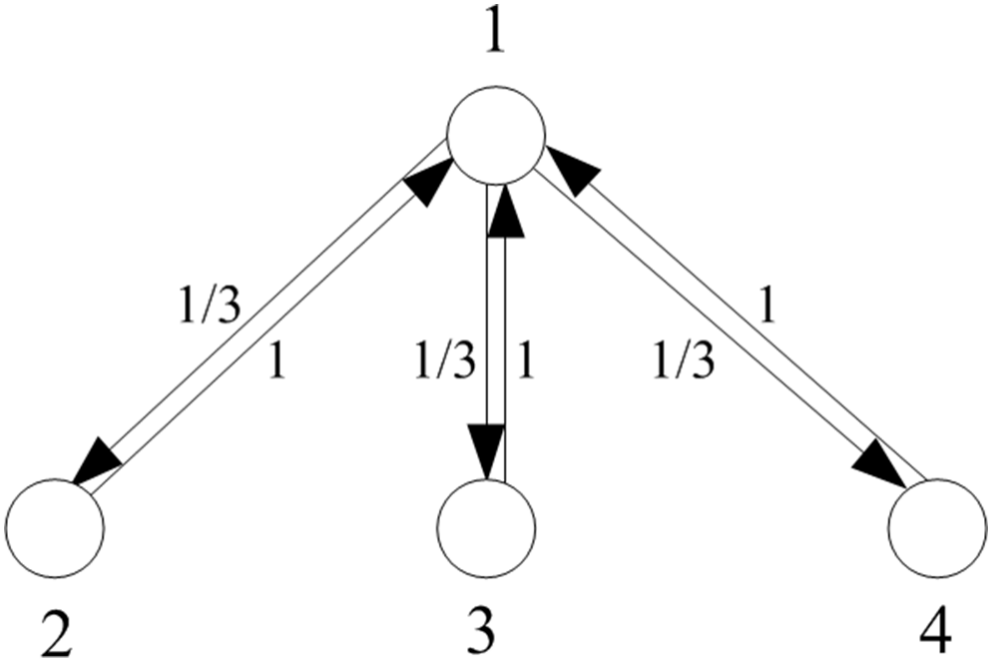
A complete oriented star S_4_ with intrinsic weights.

**Figure 2:**
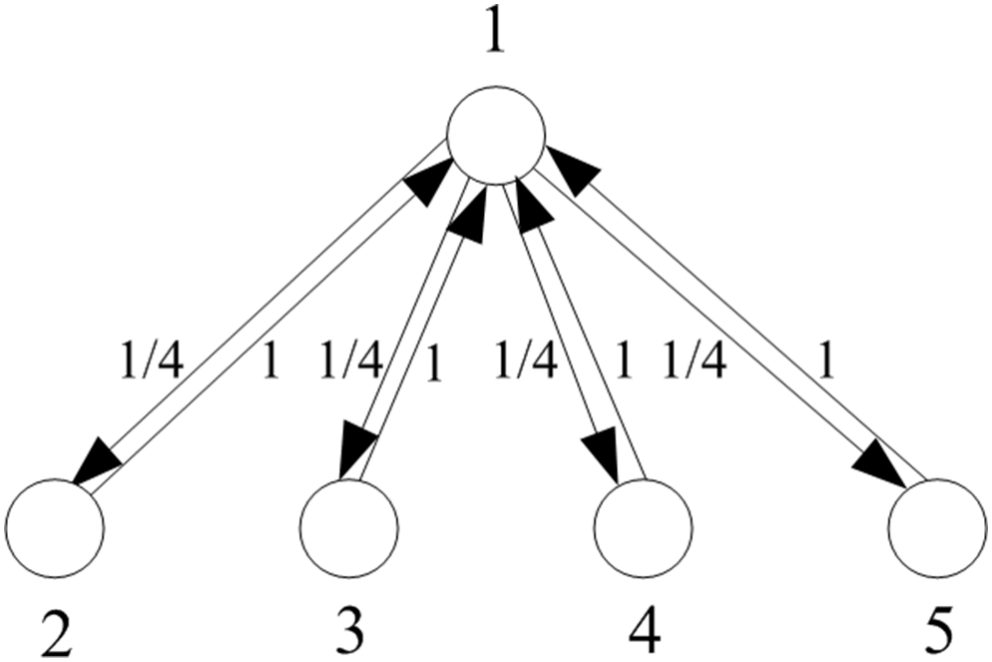
A complete oriented star S_5_ with intrinsic weights.

A *complete oriented star* (COS) of size *N*, denoted 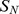, is a digraph with vertex set 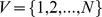 and edge set 
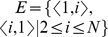
. We call vertex 1 as the *central vertex*, and vertices 2 through *N* as *peripheral vertices*. The *intrinsic weights* for 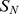 are defined as 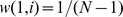 and 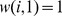 for 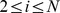. Figure 1 (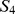) and Figure 2 (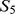) depict two COSs with intrinsic weights. In the sequel, the term “COS” means “COS with intrinsic weights”.

In a Prisoner’s Dilemma game, individuals can choose one of two strategies: *cooperation* (C) and *defection* (D), and the corresponding individuals are called *cooperator* and *defector*, respectively. The payoff matrix is given below.
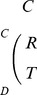
(1)where *T*, *R*, *P* and *S* stand for *temptation to defect*, *reward for mutual cooperation*, *punishment* and *sucker payoff*, respectively. It is always assumed that *T* > *R* >*P*> *S*. In this paper, we adopt the following payoff matrix suggested by Nowak and May [28]. This model preserves the essentials of the Prisoner’s Dilemma game and 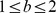 is the only tunable parameter.
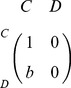
(2)


We should emphasize that our observations are not restricted to the present weak dilemma strength as the model, but remain fully valid also for the strong strength (strictly satisfying T>R>P>S).

**Figure 3:**
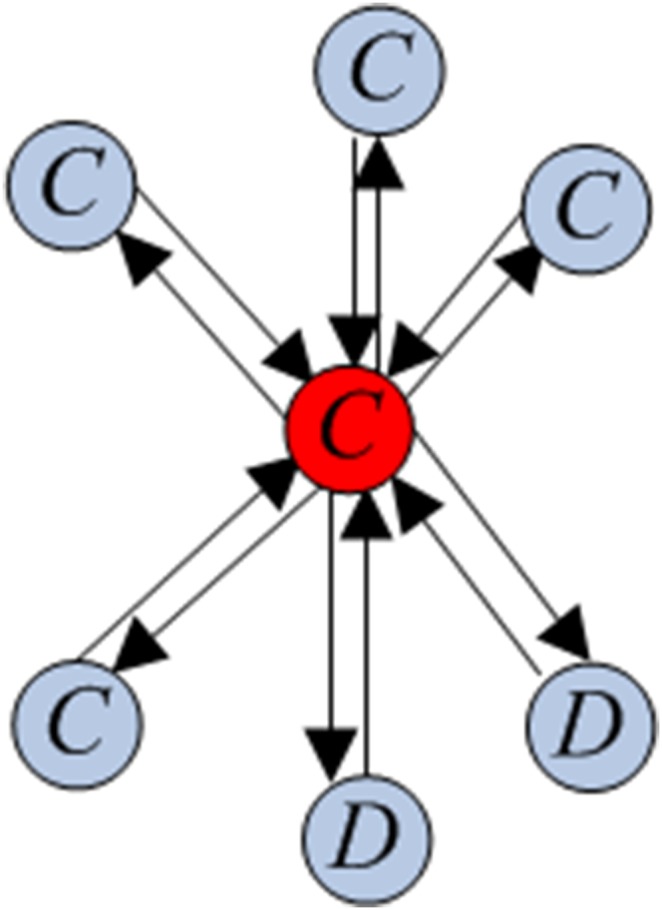
A complete oriented star with central cooperator.

**Figure 4:**
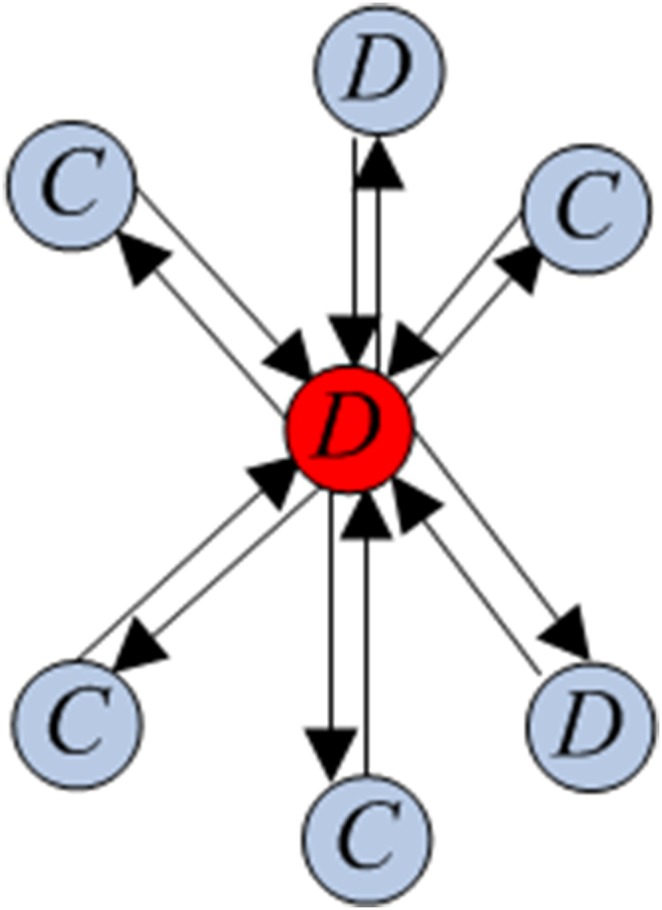
A complete oriented star with central defector.

Consider a homogeneous population with *S_N_* structure, whose individuals play a Prisoner’s Dilemma game with the payoff matrix (2). Suppose that, initially, there are *m* randomly chosen cooperators and *N* – *m* defectors. A *central cooperator* (respectively, *defector*) is a cooperator (respectively, defector) occupying the central vertex. A *peripheral cooperator* (respectively, *defector*) is a cooperator (respectively, defector) occupying a peripheral vertex. The sketches with central cooperator and central defector are given in Figure 3 and Figure 4, respectively.

Let 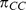 and 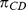 denote the mean payoffs of the central cooperator and central defector, respectively, and let 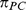 and 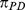 denote the payoffs of a peripheral cooperator and a peripheral defector provided the central vertex is a cooperator and defector, respectively. A straightforward calculation will give us
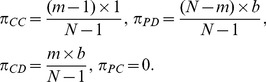
(3)


Accordingly, the payoff differences are given by
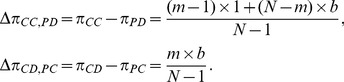
(4)


In this paper, we adopt a process based on pairwise comparison between individuals. In each step of the process, an random (focal) individual 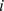 is selected for reproducing an offspring, which means an ancestor reproduces an offspring rather than one individual breaks down into two individuals, then the offspring compares its payoff 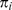 to the payoff 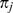 of a randomly chosen neighbor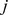, and adopts the strategy of that neighbor with probability 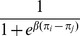 ([25], [26], [27]), where 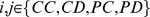
and the parameter 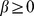, which corresponds to an inverse temperature in statistical physics, controls the intensity of selection. Small 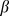 (high temperature) means that selection is almost neutral, whereas for large 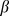 (low temperature) selection can become arbitrarily strong. This process occurs with probability 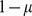; with probability 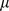 a mutation occurs, which means that the focal individual produces an offspring with random strategy, *C* or *D*.

The *m*th-order *fixation probability* on *S_N_*, denoted 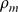, is defined as the possibility of the event that *m* cooperators could eventually take over the entire population.

## Formulas for Fixation Probabilities

In this section, we derive the formulas for the fixation probabilities on 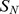.

At time *t*, the configuration of a population on 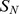 is described by a vector 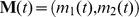, where 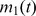  = 1 or 0 according as a cooperator occupies vertex 1or not, and 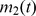 denotes the number of cooperators staying at vertices 2 through *N*. Let *m*(*t*) denote the total number of cooperators at time *t*, then 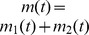. Let 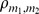 denote the probability that, starting with 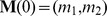, the cooperation finally fixates. For brevity, let 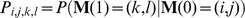 denote the conditional probability, where 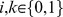 and 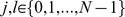. The probability of an offspring of central cooperator adopting strategy *D* (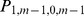) can be derived from the two sources: (1) the offspring compares its payoff to the payoff of a peripheral defector and adopts its strategy with probability 
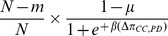
; (2) the offspring mutates into defector with probability 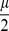. Analogously, The probability of an offspring of a selected peripheral defector adopting strategy *C* (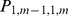) can be derived from the two sources: (1) the offspring compares its payoff to the payoff of a central cooperator and adopts its strategy with probability 
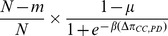
; (2) the offspring mutates into cooperator with probability 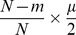. It is easy to calculate the following transition probabilities:
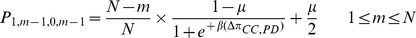
(5a)

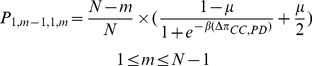
(5b)

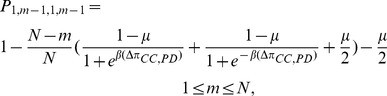
(5c)By a similar arguments, the transition probabilities 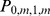 and 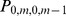 are given by the following
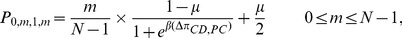
(5d)

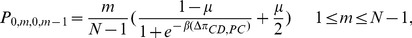
(5e)

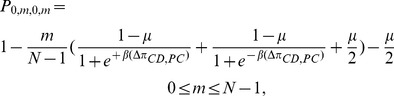
(5f)By the total probability formula we have
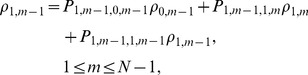
(6a)

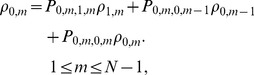
(6b)Substituting equations (5a)-(5f) into (6a) and (6b) and simplifying, we get
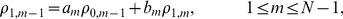
(6c)

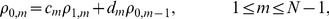
(6d)where
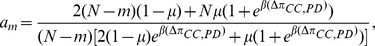


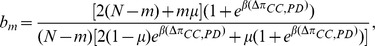


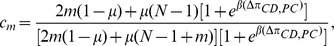


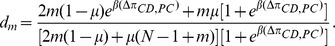
We transform equations (6c) and (6d) into linear equations:
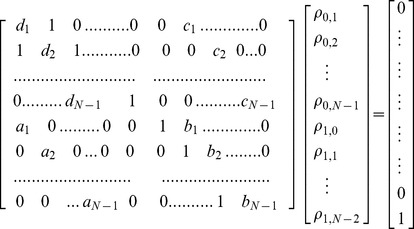
(7)According to Cramer rule, we can obtain
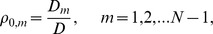


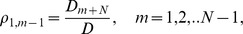


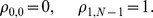
where 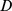 is the determinant for the first term left hand side of linear equations (7), 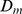 and 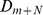 are the determinants by replacing the *m*th-column, *(m+N)*th-column in 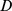 by the term on the right hand side of liner equations (7).
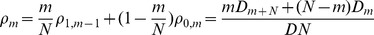
(8)


## Numerical Examples

When 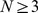, it is more difficult to investigate the properties of 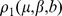 (see appendix S1). In this section, through numerical examples we investigate how the parameters affect the evolution of the Prisoner’s Dilemma game on a complete oriented star.

**Figure 5:**
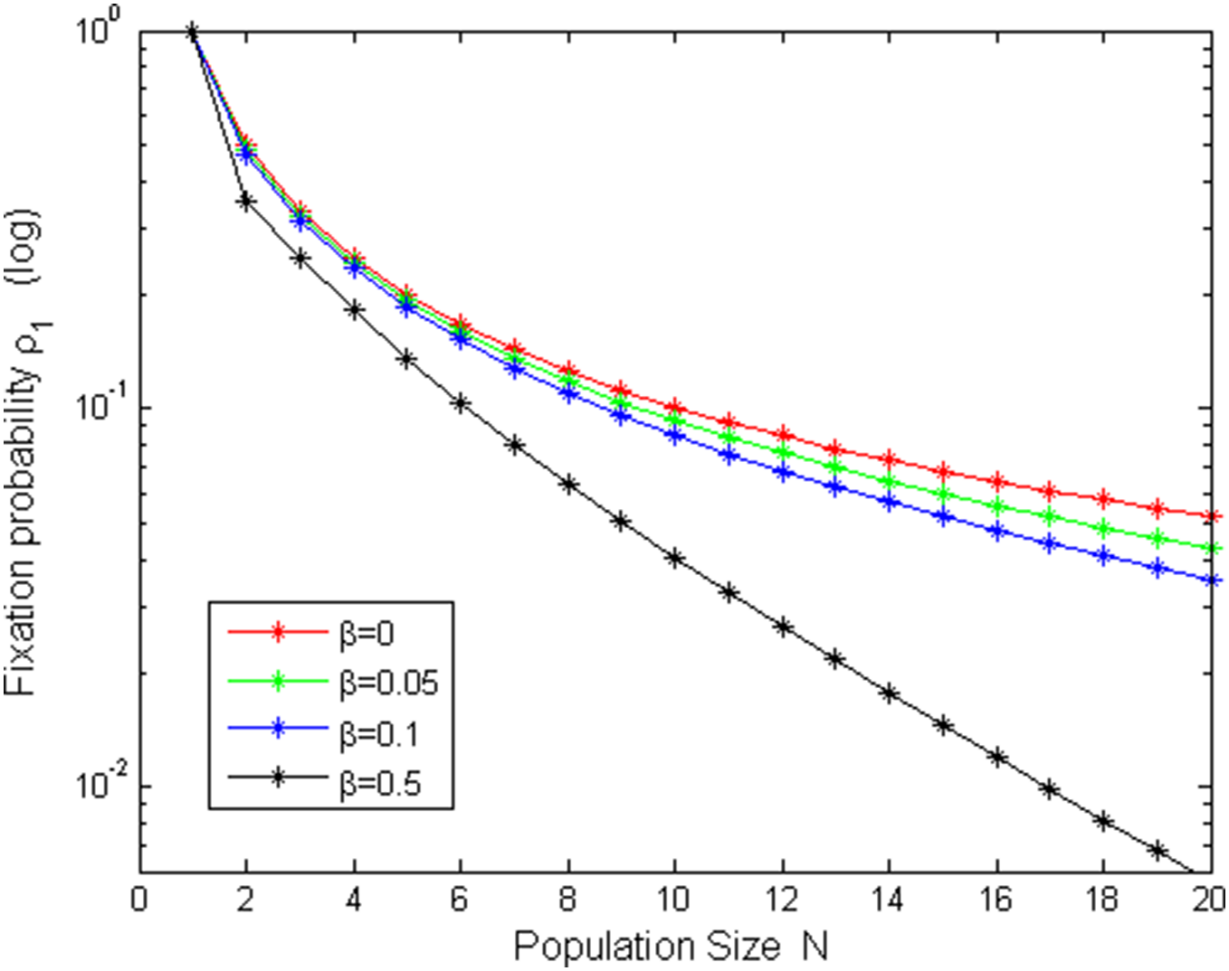
ρ_1_ vs N with µ = 1/4000, b = 1.2 and different β values.

**Figure 6:**
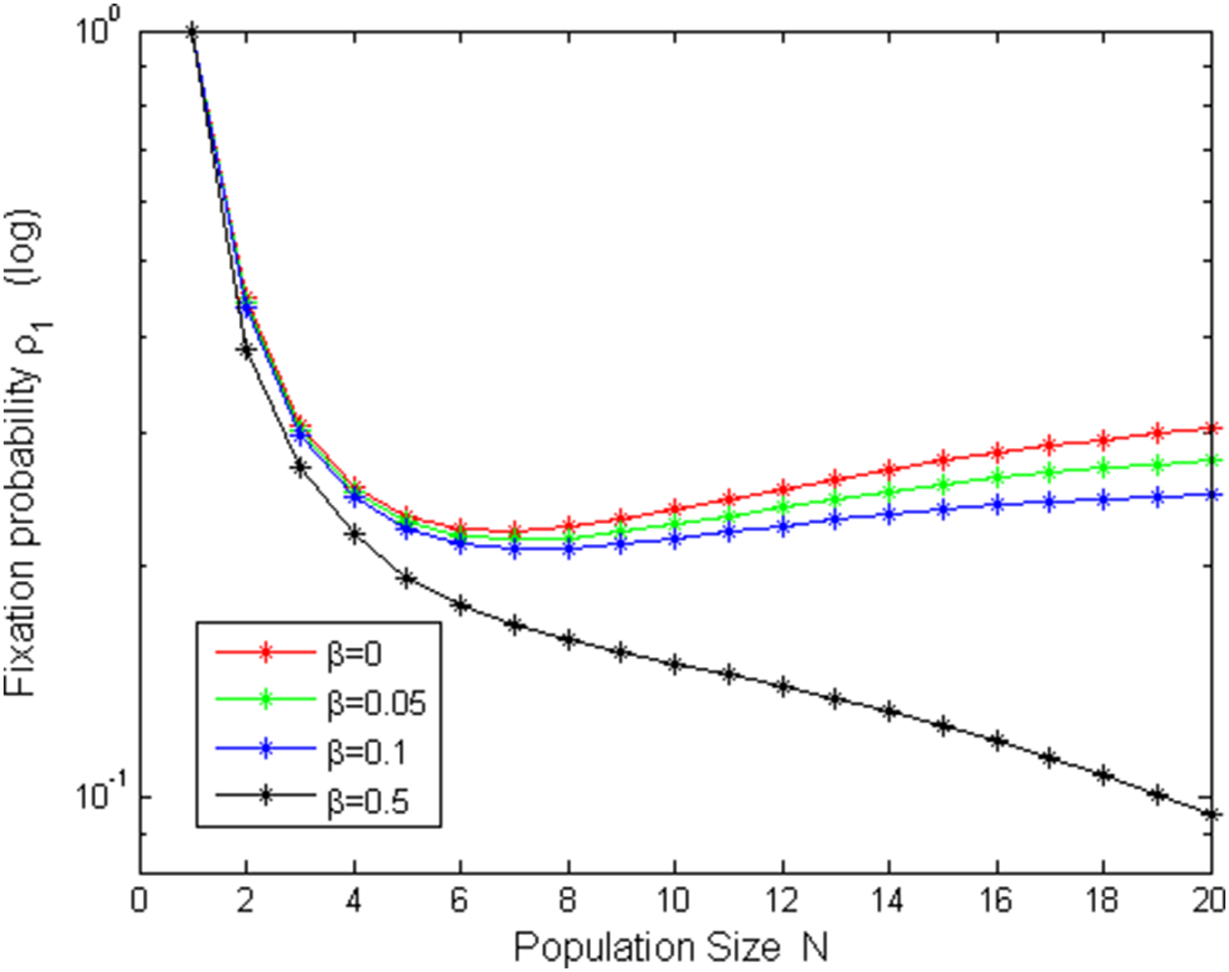
ρ_1_ vs N with µ = 0.95, b = 1.2 and different β values.

**Figure 7:**
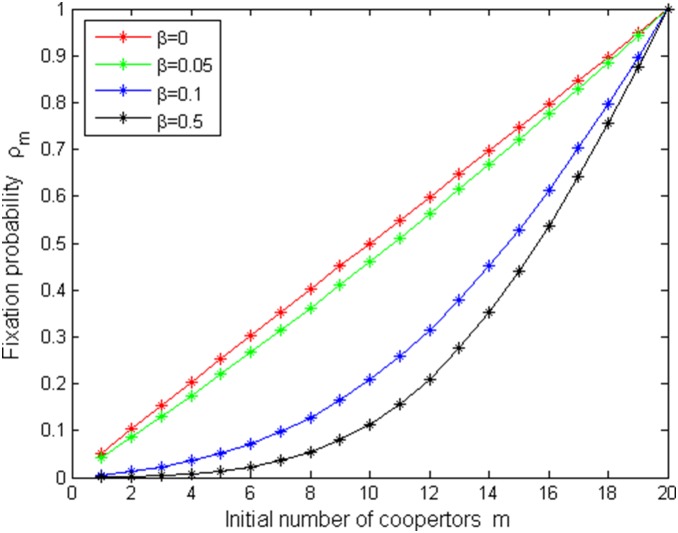
ρ_m_ vs N with µ = 1/4000, b = 1.2 and different β values.

**Figure 8:**
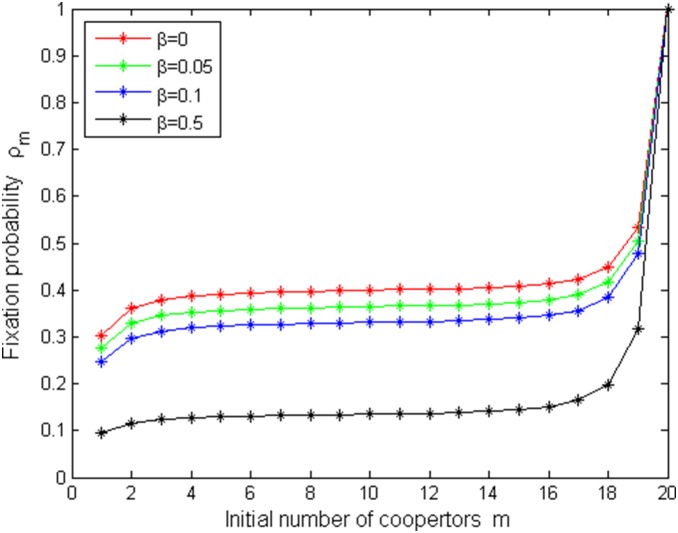
ρ_m_ vs N with µ = 0.95, b = 1.2 and different β values.

First, we will focus our attention on how fixation probability is affected by population size *N and* selection temperature 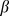 *and* initial number of cooperators *m* in given population size (let *N* = 20) *under the low mutation rates (* 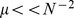 *) and high mutation rates (* 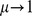 *)* ([29], [30], [31], [32], [33]). The simulations are as follows:

Figure 5–8 shows fixation probabilities in the pairwise comparison process under *the low mutation rates* and *high mutation rates* and different selection temperatures 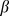. Figure 5–6 describes the first-order fixation probability 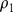 as a function of population size *N* under the different selection temperatures 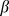. Obviously, in Figure 6, there is a slight turning of 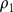 with the increment of *N* under small 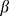 but circumstances turn out to be different in Figure 5, in which 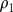 is decreasing with *N*, which accords with a fact: the larger the population size, the more difficultly to taken over it for a single cooperator with given strategy. Figure 7–8 depicts *m*th-order fixation probability 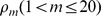 as a function of initial number of cooperators *m* under the different selection temperatures 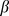. 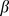 = 0 (red circle) means neutral selection and 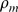 is given by the fraction of cooperators in Figure 7, which is similar as done in the evolutionary game in well-mixed populations ([26]). However, that is not the case in Figure 8 because of high mutation rates. One can see that with the increment of *m*, 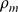 increases in Figure 7, but in Figure 8, only for a high initial number of cooperators, they have reasonable changes. 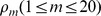 decreases with the increment of 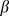 if fix *N* in Figure 7–8.

**Figure 9:**
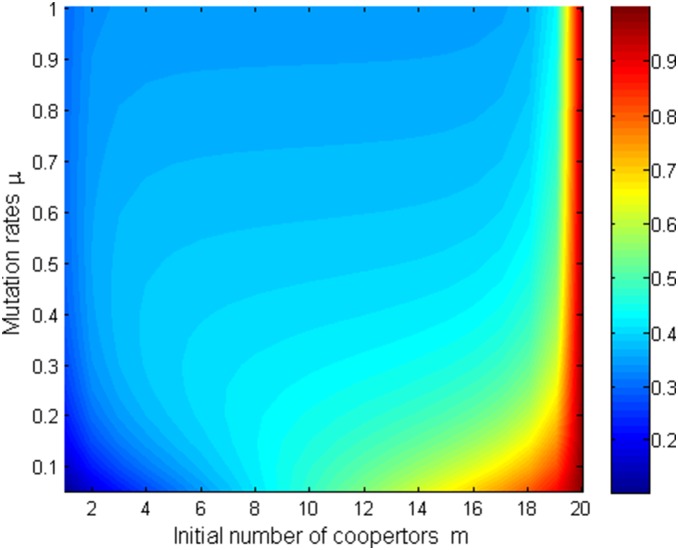
µ, m vs ρ_m_ with β = 0.01, b = 1.2.

**Figure 10:**
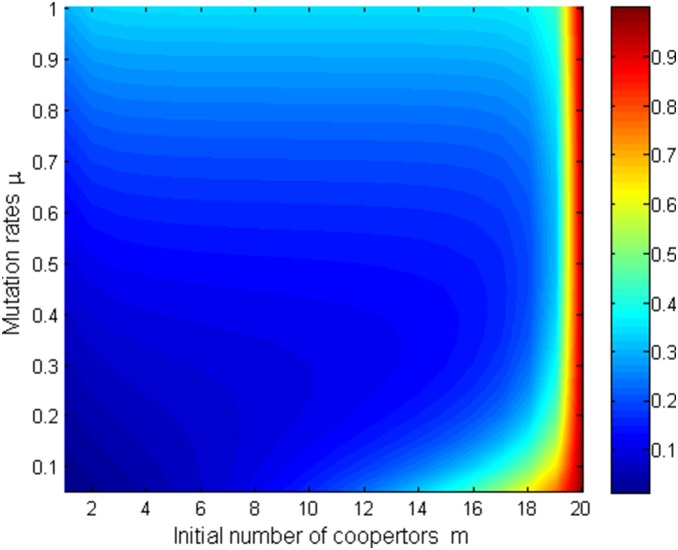
µ, m vs ρ_m_ with β = 0.5, b = 1.2.

**Figure 11:**
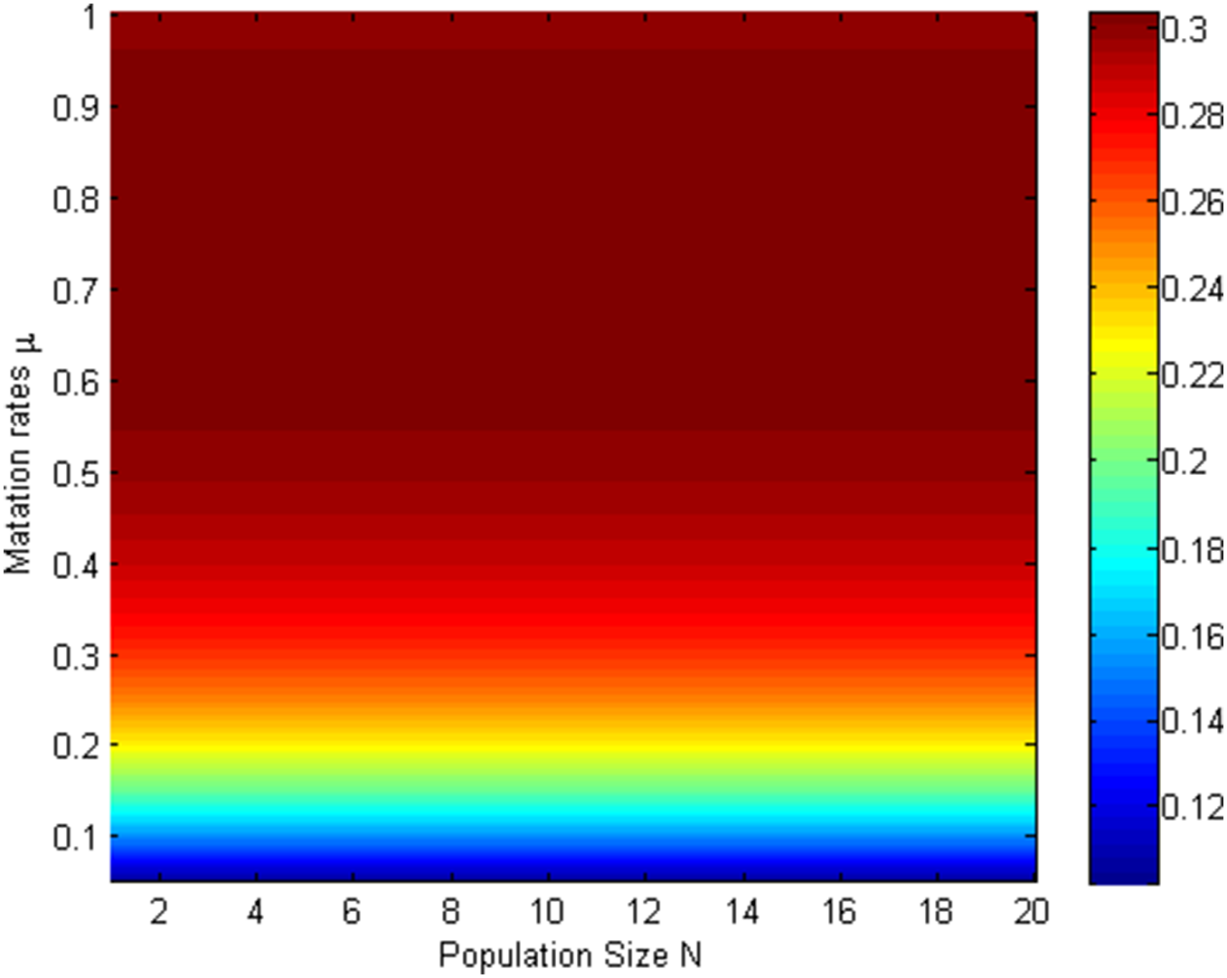
µ, m vs ρ_1_ with β = 0.01, b = 1.2.

**Figure 12:**
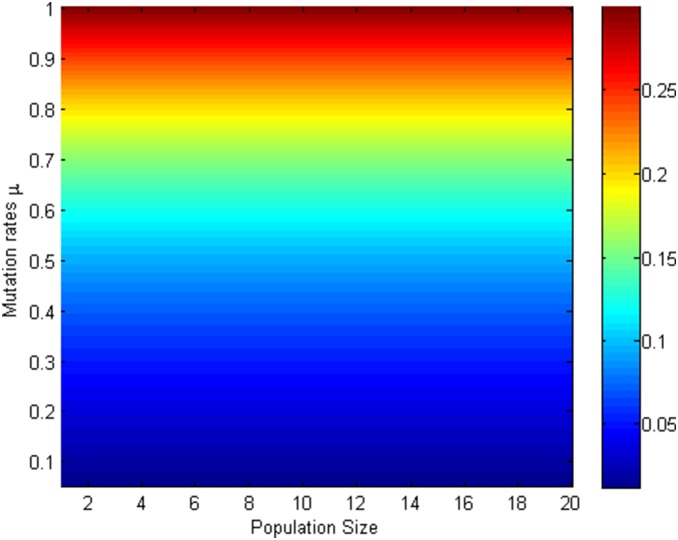
µ, m vs ρ_1_ with β = 0.5, b = 1.2.

Since Figure 5–8 shows that mutation rates 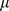 plays a key role on the fixation probabilities, to further demonstrate the effects of mutation rates 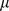, next we will investigate the relationship of 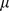, and (i) *m*th-order probability 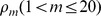 and initial number of cooperators in Figure 9 (*β* = 0.01, *b* = 1.2) and Figure10 (*β* = 0.5, *b* = 1.2) (ii) the first-order fixation probability 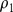 and population size in Figure 11 (*β* = 0.01, *b* = 1.2) and Figure 12 (*β* = 0.5, *b* = 1.2) under the different selection temperatures 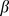. In Figure 9–10, one can see roughly that 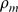 can be mainly divided into parts obviously and the corresponding parameters space are given following the order of from little to great. When m is relatively large, 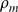 has a remarkable increment. In particular, In Figure 10, but only for a high initial number of cooperators and no matter under what values of 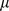, 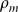 can change obviously and it is not without that, 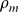 can maintain a more distinct increment. A clear description in which 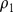 increases with the increment of 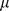 is displayed in Figure 11–12, but the degree of increment is smaller in Figure 12 than that in Figure 11.

## Concluding Remarks

In this paper, we have chosen a complete oriented star (COS) to study how the mutation rates and selection temperature and population size affects the prisoner’s dilemma game based on pairwise comparison in finite populations. A method has been derived to calculate the fixation probabilities. Then we observe and analyze effects of selection temperature and mutation rates and population size on fixation probability. We will also attempting to modify the model under study to adapt the evolution of the trustworthiness of large-scale distributed systems. It is also worth extending this work to, say, the cooperation on a pair of graphs, or on hyper graph ([34]).

## Supporting Information

Appendix S1 **The expression of first-order fixation probability for S_3.**
(DOCX)

## References

[1] Ohtsuki H, Hauert C, Lieberman E, Nowak MA (2006) A simple rule for the evolution of cooperation on graphs and social networks, Nature. 441: 502–505. 10.1038/nature04605 PMC2430087 16724065

[2] Lieberman E, Hauert C, Nowak MA (2005) Evolutionary dynamics on graphs, Nature. 433: 312–316. 10.1038/nature03204 15662424

[3] Tanimoto J (2010) The effect of assortativity by degree on emerging cooperation in a 2×2 dilemma game played on an evolutionary network, Physica A. 380: 3325–3335.

[4] Poncela J, Gomez-Gardenes J, Floría LM, Moreno Y (2007) Robustness of cooperation in the evolutionary prisoner’s dilemma on complex networks. New J. Phys. 184: 1–14.

[5] Doebeli M, Hauert C (2005) Models of cooperation based on the prisoner’s dilemma and snowdrift game. Ecol. Lett. 8: 748–766.

[6] Tanimoto J, Sagara H (2007) Relationship between dilemma occurrence and existence of a weakly dominant strategy in a two-player symmetric game. Biosystem 90(1): 105–114. 10.1016/j.biosystems.2006.07.005 17188808

[7] Gao X, Zhong W, Mei S (2013) Stochastic Evolutionary Game Dynamics and Their Selection Mechanisms. Comput. Econ. 4: 233–247.

[8] Wang Z, Kokubo S, Tanimoto J, Fukuda E, Shigaki K (2013) Insight into the so-called spatial reciprocity. Phys. Rev. E 88: 042145. 10.1103/PhysRevE.88.042145 24229153

[9] Shigaki K, Wang Z, Tanimoto J, Fukuda E (2013) Effect of Initial Fraction of Cooperators on Cooperative Behavior in Evolutionary Prisoner’s Dilemma Game. PloS One 8(11): e76942. 24244270 10.1371/journal.pone.0076942 PMC3820665

[10] Miyaji K, Tanimoto J, Wang Z, Hagishima A, Lkegaya N (2013) Direct Reciprocity in Spatial Populations Enhances R-Reciprocity as well as ST-Reciprocity. PloS One 8(8): e71961. 23951272 10.1371/journal.pone.0071961 PMC3737211

[11] Miyaji K, Wang Z, Tanimoto J, Hagishima A, Kokubo S (2013) The evolution of fairness in the coevolutionary ultimatum games. Chaos, Solitons & Fract (56) 13–18.

[12] Shigaki K, Tanimoto J, Wang Z, Kokubo S, Hagishima A (2012) et.al (2012) Referring to the social performance promotes cooperation in spatial prisoner’s dilemma games. Phys. Rev. E 86: 031141. 10.1103/PhysRevE.86.031141 23030900

[13] Wang Z, Szolnoki A, Perc M (2014) Self-organization towards optimally interdependent networks by means of coevolution. New J. Phys. 16: 033041.

[14] Qing J, Lin W, Xia CY (2014) Spontaneous Symmetry Breaking in Interdependent Networked Game, Sci. Rep (4) 4095. 10.1038/srep04095 PMC3924213 24526076

[15] Du W, Cao X, Zhao L, Hu M (2009) Evolutionary games on scale-free networks with a preferential selection mechanism. Physica A 388: 4509–4514.

[16] Li G, Jin X, Song ZH (2012) Evolutionary game on a stochastic growth network. Physica A 391: 6664–6673.

[17] Fort H (2008) on evolutionary spatial heterogeneous games. Physica A 387: 1613–1620.

[18] Tarnita CE (2009) Strategy selection in structured population, J. Theoret. Biol. 259: 570–581. 10.1016/j.jtbi.2009.03.035 PMC2710410 19358858

[19] Brewster RC, Hell P, Rizzi R (2008) Oriented star packings. J. Combin. Theor. B 98(3): 558–576.

[20] West DB (2001) Introduction to Graph Theory, Second Edition, Prentice-Hall. Inc.

[21] Szabó G, T? ke C (1998) Evolutionary Prisoner’s Dilemma game on a square lattice. Phys. Rev. E: 58–69.

[22] Chiong R, Kirley M (2012) Random mobility and the evolution of cooperation in spatial N-player iterated Prisoner’s Dilemma games. Physica A 391: 1915–3923.

[23] Li P, Duan H (2014) Robustness of cooperation on scale-free networks in the evolutionary prisoner’s game. EPL 105: 48003.

[24] Grujić J, Fosco C, Araujo L, Cuesta JA (2010) Social experiments in the mesoscale: humans playing a spatial prisoner’s dilemma. Plos One, 11. 10.1371/journal.pone.0013749 PMC2980480 21103058

[25] Hauert C, Szabó G (2005) Game theory and physics. Am. J. Phys. 73: 405–414.

[26] Traulsen A (2007) Pairwise comparison and selection temperature in evolutionary game dynamics. J. Theor. Biol. 246: 522–529. 10.1016/j.jtbi.2007.01.002 PMC2001316 17292423

[27] Blume LE (1993) The statistical mechanics of strategic interaction, Game Econ. Behav. 4: 387–424.

[28] Nowak MA, May RM (1992) Evolutionary games and spatial chaos, Nature. 359: 826–829.

[29] Nowak MA, Sasaki A, Taylor C, Fudenberg D (2004) Emergence of cooperation and evolutionary stability in finite populations. Nature 428: 646–650. 15071593 10.1038/nature02414

[30] Kondori M, Mailath M, Rob GJ (1993) Leaning mutation and long run equilibria in games, Econometrica. 61: 29–56.

[31] Taylor C, Fudenberg D, Sasaki A, Nowak MA (2004) Evolutionary game dynamics in finite populations. Bull.Math.Biol. 66 1621–1644. 10.1016/j.bulm.2004.03.004 15522348

[32] Imhof LA, Fudenberg D (2006) Imitation process with small mutations. J. Econ. Theory 131: 251–262.

[33] Antal T, Nowak MA (2009) Strategy abundance in 2×2 games for arbitrary mutation rates. J. Theor. Biol (257) 340–344. 10.1016/j.jtbi.2008.11.023 PMC2699184 19111558

[34] Paley CJ, Taraskin SN, Elliott SR (2007) Temporal and dimensional effects in evolutionary graph theory. Phys. Rev. Lett. 98: no. 9, id. 198103. 10.1103/PhysRevLett.98.098103 17359200

