# Supplementary material for: Modelling the Effects of Selection Temperature and Mutation on the Prisoner’s Dilemma Game on a Complete Oriented Star
Source: PLoS One. 2014 Oct 14;9(10):e107417. doi: 10.1371/journal.pone.0107417 (PMC4196771; doi:10.1371/journal.pone.0107417)
Supplement: Appendix S1 — The expression of first-order fixation probability for S_3. (DOCX) [file pone.0107417.s001.docx]

For $S_{3}$, using MAPLE we get
